# Discovery and Characterization of Bukakata orbivirus (*Reoviridae:Orbivirus*), a Novel Virus from a Ugandan Bat

**DOI:** 10.3390/v11030209

**Published:** 2019-03-02

**Authors:** Anna C. Fagre, Justin S. Lee, Robert M. Kityo, Nicholas A. Bergren, Eric C. Mossel, Teddy Nakayiki, Betty Nalikka, Luke Nyakarahuka, Amy T. Gilbert, Julian Kerbis Peterhans, Mary B. Crabtree, Jonathan S. Towner, Brian R. Amman, Tara K. Sealy, Amy J. Schuh, Stuart T. Nichol, Julius J. Lutwama, Barry R. Miller, Rebekah C. Kading

**Affiliations:** 1 Department of Microbiology, Immunology, and Pathology, Colorado State University, Fort Collins, CO 80523, USA; anna.fagre@colostate.edu (A.C.F.); justin.lee@colostate.edu (J.S.L.); nicholas.bergren@colostate.edu (N.A.B.); 2 Department of Zoology, Entomology and Fisheries Sciences, Makerere University, Kampala, Uganda; kityrob@gmail.com (R.M.K.); bnalikka@gmail.com (B.N.); 3 Arboviral Diseases Branch, Division of Vector-borne Diseases, Centers for Disease Control and Prevention, Fort Collins, CO 80523, USA; ilv8@cdc.gov (E.C.M.); mbcrab@gmail.com (M.B.C.); millerbr1@msn.com (B.R.M.); 4 Department of Arbovirology, Emerging, and Re-emerging Viral Infections, Uganda Virus Research Institute, Entebbe, Uganda; nakayikiteddie@yahoo.com (T.N.); nyakarahuka@gmail.com (L.N.); jjlutwama03@yahoo.com (J.J.L.); 5 Department of Biosecurity, Ecosystems and Veterinary Public Health, Makerere University, Kampala, Uganda; 6 National Wildlife Research Center, US Department of Agriculture, Animal and Plant Health Inspection Service, Wildlife Services, Fort Collins, CO 80521, USA; Amy.T.Gilbert@aphis.usda.gov; 7 College of Arts and Sciences, Roosevelt University, Collections & Research, The Field Museum of Natural History, Chicago, IL 60605, USA; jkerbis@fieldmuseum.org; 8 Viral Special Pathogens Branch, Division of High Consequence Pathogens and Pathology, Centers for Disease Control and Prevention, Atlanta, GA 30333, USA; jit8@cdc.gov (J.S.T.); cxx1@cdc.gov (B.R.A.); tss3@cdc.gov (T.K.S.); wuc2@cdc.gov (A.J.S.); Stn1@cdc.gov (S.T.N.); 9 United States Public Health Service, Commissioned Corps, Rockville, MD 20852, USA

**Keywords:** arbovirus, bat, orbivirus, next-generation sequencing, surveillance, Reoviridae

## Abstract

While serological and virological evidence documents the exposure of bats to medically-important arboviruses, their role as reservoirs or amplifying hosts is less well-characterized. We describe a novel orbivirus (*Reoviridae:Orbivirus*) isolated from an Egyptian fruit bat (*Rousettus aegyptiacus leachii*) trapped in 2013 in Uganda and named Bukakata orbivirus. This is the fifth orbivirus isolated from a bat, however genetic information had previously only been available for one bat-associated orbivirus. We performed whole-genome sequencing on Bukakata orbivirus and three other bat-associated orbiviruses (Fomede, Ife, and Japanaut) to assess their phylogenetic relationship within the genus *Orbivirus* and develop hypotheses regarding potential arthropod vectors. Replication kinetics were assessed for Bukakata orbivirus in three different vertebrate cell lines. Lastly, qRT-PCR and nested PCR were used to determine the prevalence of Bukakata orbivirus RNA in archived samples from three populations of Egyptian fruit bats and one population of cave-associated soft ticks in Uganda. Complete coding sequences were obtained for all ten segments of Fomede, Ife, and Japanaut orbiviruses and for nine of the ten segments for Bukakata orbivirus. Phylogenetic analysis placed Bukakata and Fomede in the tick-borne orbivirus clade and Ife and Japanaut within the *Culicoides*/phlebotomine sandfly orbivirus clade. Further, Bukakata and Fomede appear to be serotypes of the *Chobar Gorge virus* species. Bukakata orbivirus replicated to high titers (10^6^–10^7^ PFU/mL) in Vero, BHK-21 [C-13], and R06E (Egyptian fruit bat) cells. Preliminary screening of archived bat and tick samples do not support Bukakata orbivirus presence in these collections, however additional testing is warranted given the phylogenetic associations observed. This study provided complete coding sequence for several bat-associated orbiviruses and in vitro characterization of a bat-associated orbivirus. Our results indicate that bats may play an important role in the epidemiology of viruses in the genus *Orbivirus* and further investigation is warranted into vector-host associations and ongoing surveillance efforts.

## 1. Introduction

Serological and virological evidence documents the exposure of various East African bat species to several arboviruses including Rift Valley fever, dengue, and yellow fever virus [1,2], however, little is known about the potential role of bats as arbovirus reservoirs or potential amplifying hosts. Orbiviruses (*Reoviridae:Orbivirus*) are 10-segmented, dsRNA, vector-borne viruses that cluster phylogenetically by arthropod vector group [3]. Recent research has also reported a novel orbivirus that may represent the first recognized insect-specific virus in the genus *Orbivirus* [4]. While mostly recognized as veterinary pathogens (i.e., bluetongue virus, African horse sickness), several orbiviruses have been associated with neurologic disease in humans [5,6,7,8].

Prior to this study, four orbiviruses had been isolated from wild bats. Japanaut virus (JAPV) was isolated from the blood of a southern blossom bat (*Syconycteris crassa*) and a pool of mixed culicine mosquitoes in the Sepik District, New Guinea in 1965 [9,10]. Heramatsu orbivirus was isolated from the blood of an eastern long-fingered bat (*Myotis macrodactylus*) trapped in a mine in Heramatsu, Kagoshima, Japan in 1965 [11]. The genome of Heramatsu orbivirus (isolate KY-663) has been partially sequenced [12]. Eight isolates of Ife virus (IFEV) were isolated from the blood and organs of straw-colored fruit bats (*Eidolon helvum*) in Nigeria, Cameroon, and the Central African Republic in 1971 and 1974 [13]. Gambian pouched rats (*Cricetomys gambianus*), African grass rats (*Arvicanthis niloticus*), and domestic ruminants in Nigeria were also found to be seropositive for IFEV [14,15,16]. Fomede virus (FOMV) was isolated from the brain, liver, and spleen of a dwarf slit-faced bat (*Nycteris nana*) in Kindia, Guinea in 1978, and has been repeatedly isolated from Nycteridae bats in Guinea [17,18,19]. Additionally, serologic evidence exists for exposure of Bolivian bats (genera *Myotis* and *Noctilio*) to Matucare virus, an orbivirus isolated from *Ornithodoros* ticks in 1963 [20]. Australian fruit bats were found to be seropositive to Elsey virus, a serotype of the mosquito-borne Peruvian horse sickness virus [21].

**Figure 1:**
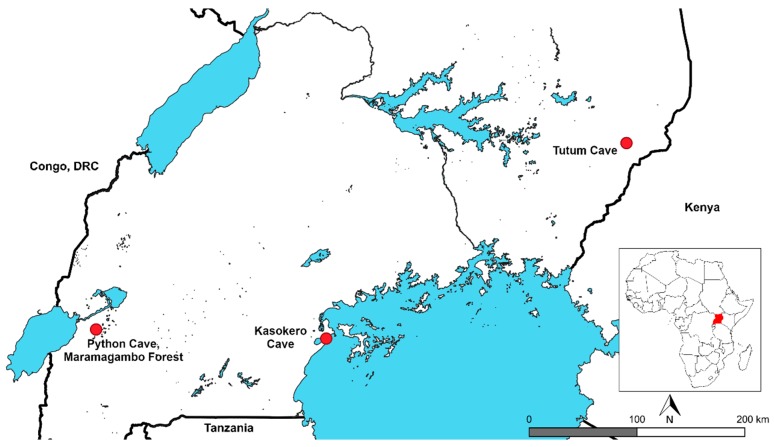
Sampling locations for bats within Uganda, 2009–2013. Capture locations for all bats tested in this study.

In 2013, a novel orbivirus, tentatively named Bukakata orbivirus (BUKV), was isolated from an Egyptian fruit bat (*Rousettus aegyptiacus leachii* (A. Smith, 1829)) (ERB) captured in Kasokero Cave, Uganda (Figure 1). This is the fifth orbivirus isolated from bats; however, aside from partial sequencing of Heramatsu, no bat-associated orbivirus has been genetically characterized. The specific aims of this project were to 1) determine the genome sequence of the four bat-associated orbiviruses without published existing genetic information (JAPV, IFEV, FOMV, & BUKV) and conduct phylogenetic analyses to ascertain their potential arthropod associations, as orbiviruses cluster phylogenetically based on their arthropod vector, 2) determine the replication kinetics of BUKV in multiple vertebrate cell types, and 3) determine the prevalence of BUKV RNA in additional archived field samples from Uganda.

## 2. Materials and Methods

### 2.1. Viruses and Cells

JAPV (MK 6357), IFEV (IbAn 57245), and FOMV (DakAnK 654) viruses were all sourced from the Arbovirus Reference Collection at the Centers for Disease Control Arbovirus Diseases Branch in Fort Collins, CO. BUKV (UGA432), isolated during this study, was deposited in the Arbovirus Reference Collection following isolation on Vero cells (African green monkey kidney epithelial cells) (ATCC CCL-81).

Vero cells and BHK-21 [C-13] cells (Syrian golden hamster kidney fibroblast) (ATCC CCL-10) were maintained in Dulbecco’s minimal essential media supplemented with 5% fetal bovine serum (FBS) (F0500-A, Atlas Biologicals, Fort Collins, CO, USA) and 1% penicillin/streptomycin (P/S) (15140122, ThermoFisher Scientific, Rockford, IL, USA). R06E cells (BEI resources, NIAID, NIH: R06E, *R. aegyptiacus leachii* (Egyptian fruit bat), Immortalized Fetal Cell Line, NR-49168) [22] were maintained in DMEM-F12 (11330032, ThermoFisher Scientific, Rockford, IL, USA) with 5% FBS and 1% P/S. R06E cells were tested and confirmed negative for contamination with Vero cells via DNAzol extraction and Sanger sequencing using Barcode of Life CO1 primers [23,24]. All cell lines were tested for presence of *Mycoplasma* spp. using PCR and were found to be negative using the ATCC Universal Mycoplasma Detection Kit (Cat. no. 30-1012K) (ATCC, Manassas, VA, USA).

### 2.2. Bat Capture and Sampling

Bats were captured from multiple locations throughout Uganda during 2011–2013 [2]. Seventy-one additional combined liver and spleen tissue RNA samples from ERBs captured previously at Maramagambo Forest in 2009 were provided for inclusion in this study (Table 1, Figure 1). All bat captures were conducted under the approval of IACUC protocols 1731AMMULX (samples from Maramagambo Forest) and 010-015 (all other samples). Bats were captured using harp traps or mist nets. Sampling locations, numbers and species captured, and blood collection and serological results from these samples are described elsewhere [2,25]. All bats were euthanized according to approved IACUC protocols in accordance with AVMA guidelines to harvest tissues for virus isolations. Tissues collected included lung, intestine, liver, spleen, and oral and fecal swabs. Tissue sections were immediately placed in cryotubes and liquid nitrogen dry shippers. Duplicate aliquots of serum and liver/spleen were analyzed first for filovirus RNA at the CDC Viral Special Pathogens laboratory in Atlanta, GA USA.

Filovirus-negative liver and spleen specimens were homogenized for virus isolation. Approximately 0.5 cm^3^ sections of tissue were mechanically homogenized in a 2.0 mL snap cap tube containing 1 mL BA1 medium (Hanks M-199 salts, 0.05 M Tris pH 7.6, 1% bovine serum albumin, 0.35 g/L sodium bicarbonate, 100 U/mL streptomycin, 1 µg/mL Fungizone) and one or two stainless steel 5 mm beads in a Qiagen mixer mill (Qiagen, Valencia, CA, USA) at 25 cycles/sec for four minutes. Homogenates were clarified by centrifugation at approximately 12,800× *g* for 8 min at 4 °C and stored at −80 °C. A 100 µL aliquot of supernatant was inoculated directly onto Vero cell monolayers, with one sample per well on a 6-well plate for virus isolation by double-overlay plaque assay [26]. A second overlay containing neutral red was added four days post infection, and plates were observed for plaques up to 10 days post infection. Cells from plaque-positive wells were harvested into 1 mL DMEM + 10% fetal bovine serum and clarified by centrifugation and the infected supernatant was stored at −80 °C. The viral RNA from plaque-positive samples was extracted from 200 µL of the supernatant and eluted into a final volume of 140 µL AE buffer using the Qiagen BioRobot EZ1 Workstation using the EZ1 Virus Mini Kit v2.0. From the infected bat, additional sections of liver, lung, intestine, and oral and fecal swabs were also processed for virus isolation as above.

### 2.3. Sequencing and Bioinformatics Analysis

Initial sequencing of the novel virus isolate and bioinformatics analysis was performed following published methods [27]. RNA was extracted from FOMV, IFEV, and JAPV virus isolates using the MagMAX Viral RNA Isolation Kit (Life Technologies, Carlsbad, CA, USA) and DNAse treated using the TURBO DNA-free™ kit (Ambion, Austin, TX, USA). Library preparation was carried out using ScriptSeq™ v2 RNA-Seq Library Preparation Kit (Epicentre Biotechnologies, Madison, WI, USA) and ScriptSeq™ Index PCR Primers (Epicentre Biotechnologies, Madison, WI, USA). Libraries were pooled and sequenced on the Illumina MiSeq using the MiSeq Reagent Kit v2 (300 cycles) (Illumina, San Diego, CA, USA). RNA was Trizol extracted from Vero cell supernatant for the novel orbivirus isolate, BUKV (UGA432), and the library was prepared using the Kapa RNA HyperPrep kit (Kapa Biosystems, Wilmington, MA, USA). The library was sequenced on the Illumina MiSeq using the MiSeq Reagent Micro Kit v2 (300 cycles) (Illumina, San Diego, CA, USA).

Low-quality bases and barcoded sequencing adapters were removed using Cutadapt, duplicate reads were collapsed using cd-hit, and host reads were filtered using Bowtie2 [28,29,30]. De novo assembly was performed using SPADES assembler and contigs were searched for homology against the NCBI Viral RefSeq database at the nucleotide (blastn) and amino acid (blastx) levels [31]. Contigs representing orbivirus genomic segments were validated by remapping the quality- and host-filtered reads using Bowtie2 [28]. The resulting alignments were visually inspected to confirm that mapping depth and base-calls were sufficient for accurately determining the sequence of each open reading frame for each genomic segment. Putative open reading frames were characterized using open reading frame (ORF) prediction (Geneious v11.1.15, Biomatters, Auckland, New Zealand) and comparing the output (ORF start/stop/length etc.) to related orbiviruses. This workflow resulted in a coding-complete consensus sequences for all ten segments of JAPV, FOMV, and IFEV, and for nine of ten segments for BUKV.

### 2.4. Phylogenetic Analysis

Genome regions of interest included the highly conserved RNA-dependent RNA polymerase (VP1), sub-core shell protein (T2 (VP2/VP3)), and the major-core surface protein (T13 (VP7)) [32,33]. Multiple alignment and phylogenetic analysis were analyzed for these three genes using the newly-derived consensus sequences obtained for the four bat-borne orbiviruses in addition to previously genetically characterized orbivirus sequences obtained from GenBank (AHSV = African horse sickness, BTV = Bluetongue virus, CGLV = Changuinola virus, CGV = Chobar Gorge virus, CORV = Corriparta virus, EEV = Equine encephalosis virus, EHDV = Epizootic hemorrhagic disease virus, EUBV = Eubenangee virus, FENGV = Fengkai virus, GIV = Great Island virus, KEMV = Kemerovo virus, LEBV = Lebombo virus, LIPV = Lipovnik virus, ORUV = Orungo virus, PALV = Palyam virus, PATAV = Pata virus, PHSV = Peruvian horse sickness virus, SVIV = Sathuvachari virus, SCRV = St. Croix River virus, SLOV = Stretch Lagoon orbivirus, TIBV = Tibet orbivirus, TILV = Tilligerry virus, TRBV = Tribec virus, UMAV = Umatilla virus, WMV = Wad Medani virus, WALV = Wallal virus, WARV = Warrego virus, YUOV = Yunnan virus) (Table S1). Putative open reading frames were translated and aligned in SeaView version 4 [34] using Clustal Omega [35], and then back-translated to nucleotide sequence. Nucleotide alignments were trimmed using TrimAl in order to remove poorly aligned regions [36].

The best-fit substitution models for nucleotide alignment and protein alignment were determined using jModelTest 2.1.10 [37,38] and ProtTest 3.4.2 [38,39], respectively. Best-fit substitution models were selected based on lowest BIC and AIC scores. The best-fit substitution models selected for amino acid multiple alignment as determined by ProtTest was LG with gamma distribution including estimation of invariant sites (LG + G + I) for VP1, T2, and T13. The best-fit substitution models selected for nucleotide multiple alignment as determined by jModelTest were generalized-time reversible with gamma distribution including estimation of variant sites (GTR + G + I) for VP1 and T2, and generalized-time reversible with gamma distribution (GTR + G) for T13. Nucleotide trees were prepared using the Bayesian Markov Chain Monte Carlo method, as implemented in MrBayes 3.2.5 [40]. The analysis was performed for five million steps, with sampling every 1000 steps and discarding the first 10% as burn-in. Amino acid maximum-likelihood trees were prepared in MEGA7 with 1000 bootstrap replicates [41,42].

### 2.5. Multi-Step Growth Curves

Confluent T-12.5 flasks of Vero cells, BHK-21 [C-13] cells, and R06E cells were inoculated with BUKV in triplicate at a MOI of 0.01. Triplicate mock-infected flasks served as negative controls. An additional flask for each cell line was prepared to count cells and ensure an accurate MOI calculation. Cells were allowed to incubate with virus for 60 min at 37 °C, and then washed with 1× phosphate buffered saline (PBS) three times prior to replacing maintenance media (DMEM with 2% FBS and 1% P/S for Vero cells and BHK-21 [C-13] cells, DMEM-F12 with 2% FBS and 1% P/S for R06E cells). Immediately, 100 µL was taken for the 0 h post-infection timepoint and proportion FBS was increased to 20% prior to freezing at −80 °C. Additional time points were collected at 12, 24, 48, 72, and 96 h post-infection. Back-titrations were performed in duplicate to confirm each inoculum was within two-fold of the desired titer. Viral quantification was assessed by plaque titration as previously described using Vero cell plaque assay [43].

### 2.6. Screening of Archived Field Samples for BUKV

In total, RNA extractions from 171 ERBs representing three populations in Uganda were available for screening [2] (Table 1, Figure 1).

RNA was extracted using the MagMax 96 total RNA isolation kit (Applied Biosystems/Ambion, Austin, TX, USA). Eluted RNA from splenic samples was tested in a quantitative reverse-transcriptase PCR (qRT-PCR) assay using TaqMan Fast Virus 1-Step Master Mix (ThermoFisher Scientific, Foster City, CA, USA). Each sample was run in duplicate using primers and probe (5′-3′) (F′: GCAGACTGTATCGCGGAAAG, R′: TAAGTTTCGCTTTCCTCCCGA, probe: CTGAAACTCGATCTCCGCAACGTTCTT) targeting the VP1 gene (RdRp) of BUKV and as single reactions using primers and probe targeting the GAPDH gene (F′: GTCGCCATCAATGACCCCTTC, R′: TTCAAGTGAGCCCCAGCC, probe: CCACCCATGGCAAGTTCAAAGGCACA) to ensure RNA integrity. Each reaction contained 5 µL splenic RNA, 5 µL TaqMan Fast Virus 1-Step Master Mix, 500 nM each primer, 250 nM probe, and 7.5 µL H_2_O. All samples were run on a QuantStudio 3 thermocycler using the following cycling parameters: 50 °C for 5 min; 95 °C for 20 s; 95 °C for 3 s, 60 °C for 30 s (40×).

As a positive control, supernatant from BUKV-infected Vero cells was 10-fold serially diluted and from each dilution, RNA was extracted using the Mag-Bind Viral DNA/RNA 96 kit (Omega Bio-Tek, Norcross, GA, USA) on the Kingfisher^®^ extraction system (Thermo Scientific, Rockford, IL, USA). Additionally, plaque assays of the dilution series were performed in duplicate on Vero cells to quantify viral titer [43]. A linear regression was performed to correlate viral titer to cutoff threshold value determined by QuantStudio™ Design & Analysis Software run on the QuantStudio™ 3 polymerase chain reaction system (Thermo Scientific, Rockford, IL, USA). The same parameters were used to screen the tick samples. Archived RNA extracted from pooled *Ornithodoros faini* soft ticks collected in Python Cave, Maramagambo forest, Uganda, were screened for BUKV RNA following the same protocol as for archived bat sample testing [44].

Samples with questionable or suspect-positive results (cycle threshold value of 35–40 or nearing the cycle threshold cutoff) using qRT-PCR were subjected to a nested PCR protocol using the Phusion HiFi Master Mix (New England BioLabs, Beverly, MA, USA) and run on a T100™ Thermal Cycler (Bio-Rad, Hercules, CA, USA). As a positive control, a synthetic oligonucleotide control was generated targeting a 406 bp region of VP1 to facilitate recognition of a false positive result by Sanger sequencing (Integrated DNA Technologies, Inc., Coralville, IA, USA) (Table S2). Cycling parameters for both Round 1 (F′: 5′-CGCTCCGTCATTGGTTTGCA-3′, R′: 5′-ATAGCTTCGCTTACGCCGGT-3′) and Round 2 (F′: 5′-TCCTTGTCTCAATCGCGCGT-3′, R′: 5′-AGCGACAAAGCCTCCACAGA-3′) were as follows: 98 °C for 30 s; 98 °C for 7 s, 68.5 °C for 15 s, 72 °C for 20 s (30×); and 72 °C for 6 min.

## 3. Results

### 3.1. Virus Isolation

Four days post-inoculation on Vero cells, plaques were visible for the spleen of bat #432 (UGA432, JCK8050, FMNH 223820) (Figure S1), a young male ERB captured in Kasokero cave in January 2013 (Table 1, Figure 1). Of the additional tissues harvested from the infected bat and tested for BUKV, only liver and spleen were positive for infectious virus by plaque assay; lung, oral and fecal swabs, and intestine were negative.

### 3.2. Genome Sequencing

Complete coding sequences were obtained for all ten segments of FOMV, IFEV, and JAPV, and for all segments besides segment 3 of BUKV. Each segment possessed one gene encoding one protein, with the exception of segments 9 and 10, which each contained a second shorter ORF similar to other orbiviruses [45,46]. The size and GC% content for each ORF, in addition to its closest match to available data on GenBank using BLASTX (predicted translation product from six reading frames) and its GenBank accession number are listed in Table 2, Table 3, Table 4 and Table 5.

### 3.3. Phylogenetic Analysis

**Figure 2:**
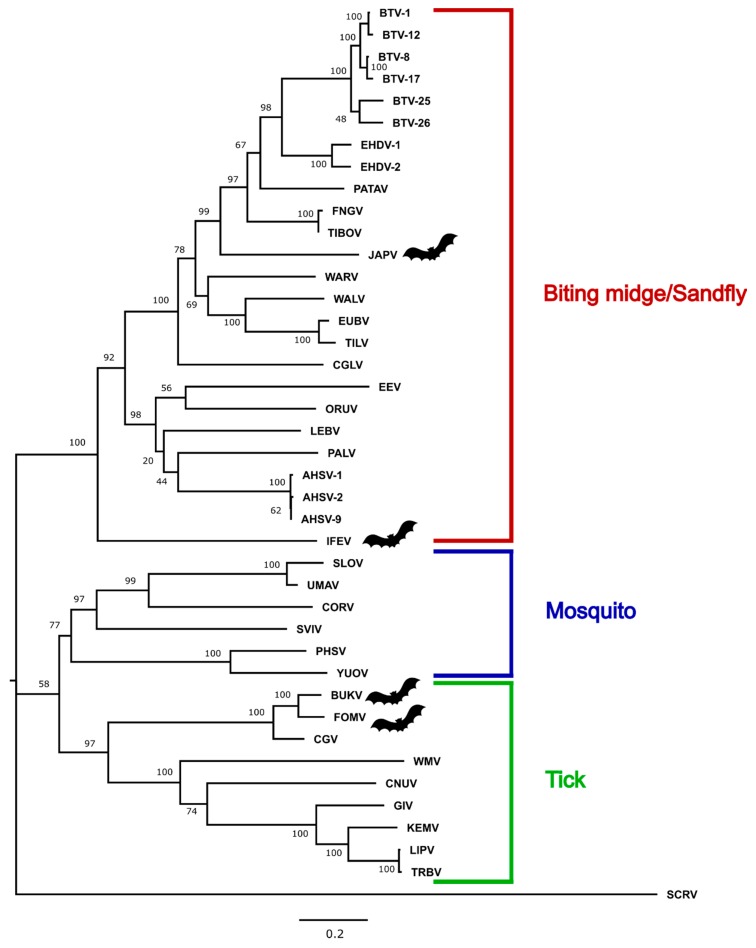
Maximum-likelihood phylogenetic tree of the viral polymerase (VP1) of selected orbiviruses (amino acid) constructed in MEGA7 using 1000 bootstrap replicates. The tree is drawn to scale, with branch lengths measured in number of substitutions per site. The tree was rooted with St. Croix River virus (SCRV). Bat symbols identify the viruses sequenced in this study. Full virus names are provided in the Materials and Methods section and accession numbers are provided in Table S1.

**Figure 3:**
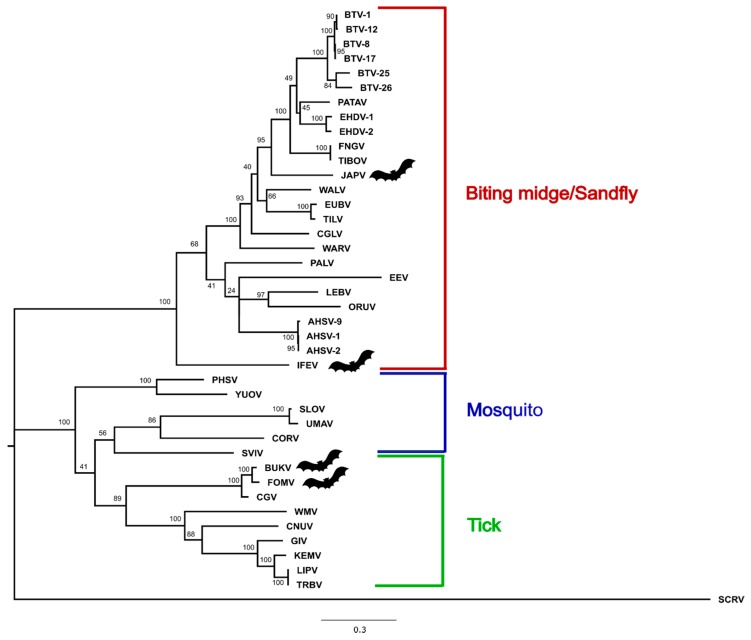
Maximum-likelihood phylogenetic tree of the sub-core shell protein (T2) of selected orbiviruses (amino acid) constructed in MEGA7 using 1000 bootstrap replicates. The tree is drawn to scale, with branch lengths measured in number of substitutions per site. The tree was rooted with St. Croix River virus (SCRV). Bat symbols identify the viruses sequenced in this study. Full virus names are provided in the Materials and Methods section and accession numbers are provided in Table S1.

**Figure 4:**
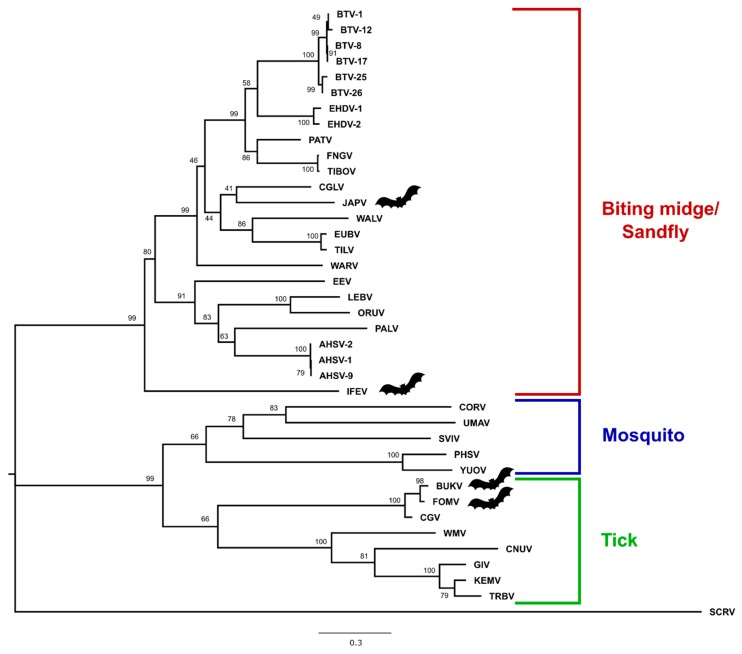
Maximum-likelihood phylogenetic tree of the outer core protein (T13) of selected orbiviruses (amino acid) constructed in MEGA7 using 1000 bootstrap replicates. The tree is drawn to scale, with branch lengths measured in number of substitutions per site. The tree was rooted with St. Croix River virus (SCRV). Bat symbols identify the viruses sequenced in this study. Full virus names are provided in the Materials and Methods section and accession numbers are provided in Table S1.

Amino-acid maximum-likelihood trees constructed in MEGA7 for VP1, T2, and T13 are provided (Figure 2, Figure 3 and Figure 4), as are nucleotide phylogenetic trees estimated using the MCMC method in MrBayes (Supplemental Figures S2–S4). Phylogenetic tree topology consistently places BUKV and FOMV viruses with the tick-borne orbiviruses and JAPV and IFEV with the *Culicoides*/sandfly-borne orbiviruses.

**Figure 5:**
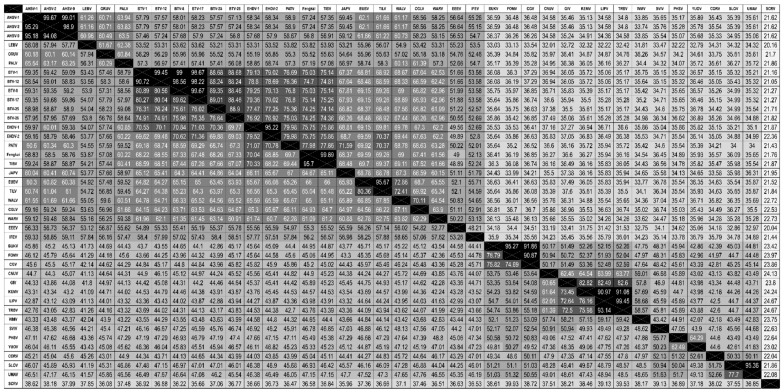
Matrix of T2 pairwise identity percentages of nucleotide (lower left) and amino acid (upper right) between members of the genus *Orbivirus*.

Pairwise distances generated in Geneious using MAFFT for both nucleotide and amino acids are listed for each of the four newly-sequenced orbiviruses in addition to the other previously-sequenced orbiviruses obtained from Genbank in Figure 5 (T2), Figure S5 (VP1), and Figure S6 (T13).

### 3.4. Growth Curves

**Figure 6:**
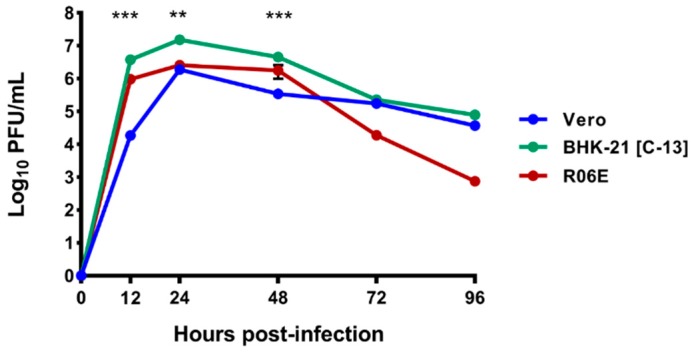
Comparison of growth kinetics of BUKV in Vero, BHK-21 [C-13], and R06E cells when infected at MOI 0.01. Means and SEs from three independent replicates are shown. Statistics were performed using a two-way ANOVA with Tukey’s correction at each time point. *p*-values: 12 hpi: <0.0001 (Vero vs. BHK-21 [C-13]), 0.0328 (Vero vs. R06E), <0.0001 (BHK-21 [C-13] vs. R06E; 24 hpi: <0.0001 (Vero vs. BHK-21 [C-13]), <0.0001 (BHK-21 [C-13] vs. R06E; 48hpi: <0.0001 (Vero vs. BHK-21 [C-13]), 0.0009 (Vero vs. R06E), <0.0001 (BHK-21 [C-13] vs. R06E). Asterisks in figure indicate the number of two-way comparisons that were significant (*p* < 0.05) at each timepoint.

Propagation of BUKV was successful in all three mammalian cell lines tested (Vero, BHK-21 [C-13], and R06E cells) with viral titers peaking at 24 hpi in all three cell lines, and the highest titer achieved in BHK-21 [C-13] cells (1.6 × 10^7^ PFU/mL at 24 hpi) (Figure 6). Cytopathic effect was detected in all three cell lines between 12 and 24 hpi.

### 3.5. Testing of Additional Bat and Tick Samples

None of the 171 bat samples screened via qRT-PCR resulted in amplification of BUKV VP1 RNA, though six were suspected or weak positive. None of the six suspect-positive bat samples were confirmed positive for BUKV RNA by nested PCR. Of the 171 samples, GAPDH RNA was successfully amplified from 86% (147/171) confirming RNA integrity. Of the 513 tick pools tested, 16s rRNA internal extraction control was amplified from 485, and none were positive for BUKV VP1 RNA.

## 4. Discussion

Bats are known to host a number of emerging zoonotic viruses highly pathogenic to humans in the absence of overt pathology within the bat host. Limited information exists, however, about the ability of bat species to harbor and transmit medically important arboviruses. Despite extensive serologic evidence suggestive of exposure of multiple bat species to arboviruses from different families (Flaviviridae, Togaviridae, and Bunyavirales), few studies have resulted in the isolation of arboviruses from wild-caught bats. Molecular and in vivo characterization of novel viruses isolated from wild-caught bats, in addition to enhanced surveillance efforts targeting suspected hosts helps clarify the role of bats as reservoirs for emerging arboviruses.

This study provides complete coding sequences of three previously uncharacterized orbiviruses isolated from bats (JAPV, IFEV, and FOMV) and one novel bat-associated orbivirus, BUKV, isolated from an ERB. Phylogenetic analyses place BUKV and FOMV in the tick-borne orbivirus clade, and IFEV and JAPV cluster with the *Culicoides*/sandfly-borne orbiviruses. This study also provides documentation for in vitro propagation of a bat-associated orbivirus in a number of different vertebrate cell lines, one of which was derived from the ERB. The screening of additional archived bat and tick samples resulted in negative findings, but reflects a critical step in the investigation process into the host-vector relationships supported by phylogenetic analyses.

Of the three segments analyzed, the topology of the phylogenetic analysis consistently placed BUKV and FOMV within the tick-borne orbivirus subclade along with Chobar Gorge virus (CGV). Past studies indicated that FOMV is a serotype of the *Chobar Gorge virus* species based on results of complement fixation, and it is considered as such by the International Committee on the Taxonomy of Viruses [3,47,48]. This is consistent with past isolations of FOMV in field-caught Ixodid ticks [17]. CGV has been isolated from *Ornithidoros* spp. ticks in Nepal, and antibodies have been detected in humans and domestic ruminant species in the same region [49]. Additionally, the clustering of BUKV, FOMV, and CGV within the same subclade of tick-borne orbiviruses and high degree of nucleotide and amino acid similarity regardless of protein analyzed suggests they are three different serotypes of the same species Attoui et al. suggested that the amino acid identity for T2 of <91% should be the criteria for designating a species within the genus Orbivirus [50]. According to that criterion BUKV and FOMV viruses are on the border for consideration as new species. BUKV possesses 95.27% amino acid similarity to FOMV and 91.56% similarity to CGV, and Fomede possesses 90.87% amino acid similarity to CGV. Bukakata and Fomede viruses may be ecologically unique from Chobar Gorge in having been isolated from bats, however it is not known whether or not Chobar Gorge virus is also found in bats. Further characterization into the evolutionary relationship of these bat-associated and potentially tick-borne orbiviruses should involve exploration into in vitro growth kinetics in invertebrate cell lines in addition to the potential for serologic cross-reactivity and in vitro reassortment potential.

JAPV and IFEV cluster with the *Culicoides*/sandfly-borne orbivirus clade and have not yet been approved as species of orbiviruses, but novel genetic sequence obtained during this study indicates that their listing should be revised. Neither JAPV nor IFEV possess the requisite >76% nucleotide identity to any other orbivirus in their conserved T2 gene, indicating they are each their own individual species [3] (Figure 5). In the analysis of all three segments, IFEV is very distantly related to all other orbiviruses and may represent its own species due to the low level of nucleotide (maximum 55.7%) and amino acid similarity (maximum 59.3%) to any other orbivirus when analyzing the gene encoding sub-core shell T2 protein (Figure 5). Interestingly, the BLASTX results for IFEV virus segments reveal that it is most closely related to Heramatsu orbivirus. Heramatsu virus was obtained from a Japanese eastern long-fingered bat in 1965 and was partially sequenced in 2013 [11,12]. However, due to lack of complete genome information and access to an archive isolate, this virus was not included in our phylogenetic analyses.

Due to their segmented genome, orbiviruses are known to undergo reassortment during co-infection [51,52]. Comparing the placement of JAPV within the VP1 and T2 phylogenies to its placement in the T13 phylogeny suggests that it may have undergone reassortment; however, definitive conclusions surrounding its potential as a reassortant virus are difficult to make due to low bootstrap values and posterior probabilities (Figure 2, Figure 3 and Figure 4, Figures S2–S4). Interestingly, our phylogenetic analyses indicate JAPV clusters with the *Culicoides*/sandfly-borne orbiviruses, though it was isolated from a pool of Culicine mosquitoes in New Guinea [10]. Further investigation is required to better characterize potential vector-host associations for JAPV and its potential as a reassortant orbivirus.

BUKV grew to high titers in all three vertebrate cell lines in which multi-step growth curves were conducted. Two of these cell lines, Vero cells and BHK-21 [C-13] cells, are deficient for the interferon pathway, while the R06E pathway has an intact interferon response [53]. The Type I interferon response is the first line of antiviral defense in the mammalian immune system [54]. Interestingly, viral titers in interferon-competent R06E cells were comparable to interferon-deficient BHK-21 [C13] and Vero cells (Figure 6). The immune system of some bat species is highly unique in its constitutive expression of IFN-α [55]. A recent study by Pavlovich and colleagues indicate that unlike *Pteropus alecto*, transcriptomic analysis of the ERB does not provide evidence of constitutive interferon expression [56]. Analysis of interferon expression over the course of infection in bat cells and other interferon-competent vertebrate lines would be an informative way to analyze the presence of this constitutive expression in existing bat cell lines.

None of the 171 bat samples or 513 tick pools tested positive for BUKV viral nucleic acid. However, GAPDH mRNA was tested for each bat sample and samples for which amplification of the GAPDH was not obtained were not included in the denominator of the total tested samples. While all samples were negative for BUKV RNA, some of the bat samples had very high CT values or were nearing the cycle threshold and as such, were considered to be suspect and subjected to a nested PCR protocol. The six suspect samples tested using this nested PCR were also confirmed negative. Samples types screened (spleen and/or liver) are consistent with the organs from which the virus was originally isolated. Testing additional bat species for viral RNA could yield additional information on the circulation of this virus.

While this study provides valuable information regarding potential vector-host associations among the orbiviruses, limitations restrict certain conclusions. Each orbivirus segment contains 1–2 genes, with untranslated regions on either end of the ORF. Due to decreased coverage at the ends of the reads, variable coverage was achieved throughout the length of each segment and only the complete coding genome of IFEV, JAPV, and FOMV were obtained. The coding complete sequence of segments 1–2 and 4–10 were obtained for BUKV but due to low coverage at the 5′ end of segment 3, the start codon was not obtained (Table 2). Individual orbivirus species possess conserved 5′ UTR and 3′ UTR terminal sequences and as such, higher coverage in the untranslated regions would have provided additional information surrounding level of relatedness between these and previously sequenced orbiviruses [3]. Field surveillance efforts were opportunistic and retrospective, and only ERB RNA was tested. The testing of additional bat species from nearby geographic areas sharing similar ecological habitats would provide additional information surrounding vertebrate host range. The tick pools tested were also opportunistic and retrospective, and originated in Python Cave, a cave with analogous ecological characteristics to Kasokero Cave, where BUKV was isolated, yet 213 km away (Table 1, Figure 1).

## 5. Conclusions

This study provided coding of the complete genome sequence on three bat-associated orbiviruses that have been discovered to date, and coding the complete sequence for nine of ten segments for a newly isolated bat-associated orbivirus. Further, in vitro replication kinetics were described in three vertebrate cell lines. From the phylogenetic analyses, inference regarding potential arthropod vector associations can be drawn regarding transmission of bat-associated orbiviruses and supports ticks as a potential vector of BUKV. While the archived bat and tick samples tested for BUKV RNA were negative, this study provides a strong framework for comprehensive viral characterization, including initial discovery, in vitro characterization, and the screening of samples collected from the potential vertebrate host and the purported invertebrate vector. The isolation of five orbiviruses from distinct bat species located across space and time and phylogenetically-associated with different arthropod vectors indicates a strong association between orbiviruses and bats, and further investigation into the public health impact of these orbiviruses is warranted.

## Figures and Tables

**Table 1:** Sampling locations and dates for Egyptian fruit bats (*R. aegyptiacus leachii*) screened for BUKV RNA [2].

Location	Latitude	Longitude	*n*	Tissue Type	Date Collected
Python Cave, Maramagambo Forest	−0.26667	30.05000	71	Liver and spleen	Nov 09
Kasokero cave	−0.34214	31.96627	56	Spleen	Jan 13
Tutum cave	1.28333	34.46667	45	Spleen	Feb 12

**Table 2:** Genetic analysis of BUKV genome segments, predicted ORFs, and predicted proteins.

Seg.	Protein Encoded	ORF Length (nt)	Pred. Protein Length	%GC Content (ORF)	Top Blastx (ORF) [Nearest Virus ^1^], Accession, % Pairwise Identity	Accession #
1	VP1	3855	1285	51.9	VP1 (Pol) [CGV], YP_009158901, 83.6%	MK359215
2	VP2 (T2)	2730	2730	52.3	VP2 (T2) [CGV], YP_009158902, 91.7%	MK359216
3	VP3 (Cap)	N/A ^2^	N/A ^2^	N/A ^2^	VP3 (Cap) [CGV], YP)009158903, 74.5%	MK359217
4	VP4 (OC1)	1761	587	50.9	VP4 (OC1) [CGV], YP_009158904, 60.1%	MK359218
5	NS1 (TuP)	1575	525	55.3	NS1 (TuP) [CGV], YP_009158905, 80.2%	MK359219
6	VP5 (OC2)	1608	536	53.7	VP5 (OC2) [CGV], YP_009158906, 79.8%	MK359220
7	VP7 (T13)	1068	356	54.1	VP7 (T13) [CGV], YP_009158907, 88.2%	MK359222
8	NS2 (ViP)	1110	370	54.6	NS2 (ViP) [CGV], YP_009158908, 76.7%	MK359221
9	VP6 (Hel)	1041	347	53.2	VP6 (Hel) [CGV], YP_009158909, 54.3%	MK359223
	NS4	717	239	54.4	VP6 (Hel) [CGV], YP_009158909, 54.3%	
10	NS3	621	207	52.3	NS3 [CGV], YP_009158911, 90.3%	MK359224
	NS3a	579	193	52.0	NS3a [CGV], YP_009158912, 90.1%	

^1^ CGV = Chobar Gorge virus. ^2^ Partial ORF provided as sequencing coverage at 5′ end was too low to obtain start codon.

**Table 3:** Genetic analysis of FOMV genome segments, predicted ORFs, and predicted proteins.

Seg.	Protein Encoded	ORF Length (nt)	Pred. Protein Length	%GC Content (ORF)	Top Blastx (ORF) [Nearest Virus ^1^], Accession, % Pairwise Identity	Accession #
1	VP1	3855	1285	52.7	VP1 (Pol) [CGV], YP_009158901, 82.8%	MK359225
2	VP2 (T2)	2730	910	50.9	VP2 (T2) [CGV], YP_009158902, 90.9%	MK359226
3	VP3 (Cap)	1908	636	53.7	VP3 (Cap) [CGV], YP_009158903, 71.4%	MK359227
4	VP4 (OC1)	1761	587	50.3	VP4 (OC1) [CGV], YP_009158904, 69.3%	MK359228
5	NS1 (TuP)	1575	525	55.7	NS1 (TuP) [CGV], YP_009158905, 78.6%	MK359229
6	VP5 (OC2)	1608	536	54.7	VP5 (OC2) [CGV], YP_009158906, 83.2%	MK359230
7	VP7 (T13)	1068	356	53.2	VP7 (T13) [CGV], YP_009158907, 89.0%	MK359232
8	NS2 (ViP)	1110	370	56.0	NS2 (ViP) [CGV], YP_009158908, 77.2%	MK359231
9	VP6 (Hel)	1041	347	55.1	N/A ^2^	MK359233
	NS4	717	239	55.2	NS4 [CGV], YP_009158910, 60.2%	
10	NS3	621	207	53.9	NS3 [CGV], YP_009158911, 88.8%	MK359234
	NS3a	570	190	53.5	NS3a [CGV], YP_009158912, 89.4%	

^1^ CGV = Chobar Gorge virus. ^2^ No blastx matches.

**Table 4:** Genetic analysis of JAPV genome segments, predicted ORFs, and predicted proteins.

Seg.	Protein Encoded	ORF Length (nt)	Pred. Protein Length	%GC Content (ORF)	Top Blastx (ORF) [Nearest Virus ^1^], Accession, % Pairwise Identity	Accession #
1	VP1	3900	1300	38.3	VP1 [TIBOV], APT68074, 66.3%	MK359235
2	VP2 (OC1)	3636	1212	39.2	VP2 (OC1) [BTV-2], CAO79540, 22.1%	MK359236
3	VP3 (T2)	2703	901	40.9	VP3 (T2) [PATV], AFH41521, 72.0%	MK359237
4	VP4 (Cap)	1932	644	40.7	VP4 (Cap) [BTV-12], ASV51737, 57.6%	MK359238
5	NS1 (TuP)	1662	554	44.8	NS1 (TuP) [EHDV-7], AIY25176, 30.3%	MK359239
6	VP5 (OC2)	1590	530	43.1	VP5 (OC2) [CGLV], AGZ91948, 54.0%	MK359240
7	VP7 (T13)	1053	351	44.5	VP7 (T13) [CGLV], YP_008719923, 57.1%	MK359241
8	NS2 (ViP)	1011	337	43.8	NS2 (ViP) [CGLV], AGZ91980, 42.6%	MK359242
9	VP6 (Hel)	804	268	44.7	VP6 (Hel) [WALV], YP_008658421, 32.7%	MK359243
	NS4	234	78	48.7	N/A ^2^	
10	NS3	786	262	43.6	NS3 [TILV], AFH41508, 39.1%	MK359244
	NS3a	642	214	44.5	NS3 [TILV], AFH41508, 39.1%	

^1^ BTV = Bluetongue virus, CGLV = Changuinola virus, EHDV = Epizootic hemorrhagic disease virus, PATV = Pata virus, TIBOV = Tibet virus, TILV = Tilligerry virus, WALV = Wallal virus. ^2^ No blastx matches.

**Table 5:** Genetic analysis of IFEV genome segments, predicted ORFs, and predicted proteins.

Seg.	Protein Encoded	ORF Length (nt)	Pred. Protein Length	%GC (ORF)	Top Blastx (ORF) [Nearest Virus ^1^], Accession, % Pairwise Identity	Accession #
1	VP1	3900	1300	39.7	VP1 [Heramatsu], AGZ62525, 62.0%	MK359245
2	VP3 (T2)	2685	895	42.6	VP3 (T2) [Heramatsu], AGZ62528, 61.9%	MK359246
3	VP2 (OC1)	2523	841	41.7	VP2 (OC1) [Heramatsu], AGZ62527, 29.1%	MK359247
4	VP4 (Cap)	1902	634	42.0	VP4 (Cap) partial [Heramatsu], AGZ62529, 52.7%	MK359248
5	NS1 (TuP)	1581	527	44.7	NS1 (TuP) [LEBV], YP_009507714, 32.0%	MK359249
6	VP5 (OC2)	1569	523	43.0	VP5 (OC2) [CGLV], AGZ91955, 46.8%	MK359250
7	VP7 (T13)	1047	349	46.2	VP7 (T13) [WALV], YP_008658420, 41.7%	MK359251
8	NS2 (ViP)	990	330	44.1	NS2 (ViP) [Heramatsu], AGZ62533, 40.5%	MK359252
9	VP6 (Hel)	768	256	47.5	VP6 (Hel) partial [Heramatsu], AGZ62534, 34.7%	MK359253
	NS4	240	80	53.8	N/A ^2^	
10	NS3	612	204	46.6	NS3 [Heramatsu], AGZ62526, 47.1%	MK359254
	NS3a	561	187	46.3	NS3 [Heramatsu], AGZ62526, 47.1%	

^1^ CGLV = Changuinola virus, LEBV = Lebombo virus, WALV = Wallal virus. ^2^ No blastx matches.

## Supplementary Materials

The following are available online at https://www.mdpi.com/1999-4915/11/3/209/s1, Figure S1. Image of BUKV plaque on Vero cells derived from spleen homogenate from bat UGA432, Figure S2. Bayesian phylogenetic tree of the viral polymerase gene (VP1) of selected orbiviruses (nucleotide), Figure S3. Bayesian phylogenetic tree of the gene encoding sub-core shell (T2) of selected orbiviruses (nucleotide), Figure S4. Bayesian phylogenetic tree of the gene encoding the outer core protein (T13) of selected orbiviruses (nucleotide), Figure S5. Matrix of VP1 pairwise identity percentages, Figure S6. Matrix of T13 pairwise identity percentages, Table S1. Sequences used in phylogenetic analyses of bat-associated orbiviruses, Table S2. Sequence of BUKV VP1 synthetic positive amplification control.

## References

[1] Calisher C.H., Childs J.E., Field H.E., Holmes K.V., Schountz T. (2006). Bats: Important reservoir hosts of emerging viruses. Clin. Microbiol. Rev..

[2] Kading R.C., Kityo R.M., Mossel E.C., Borland E.M., Nakayiki T., Nalikka B., Nyakarahuka L., Ledermann J.P., Panella N.A., Gilbert A.T. (2018). Neutralizing antibodies against flaviviruses, Babanki virus, and Rift Valley fever virus in Ugandan bats. Infect. Ecol. Epidemiol..

[3] Attoui H., Mertens P., Becnel J., Belaganahalli S., Bergoin M., Brussaard C., Chappell J., Ciarlet M., del Vas M., Dermody T. (2011). Orbiviruses, Reoviridae. Virus Taxon. Ninth Rep. Int. Comm. Taxon. Viruses.

[4] Harrison J., Warrilow D., McLean B., Watterson D., O’Brien C., Colmant A., Johansen C., Barnard R., Hall-Mendelin S., Davis S. (2016). A new orbivirus isolated from mosquitoes in North-Western Australia shows antigenic and genetic similarity to Corriparta virus but does not replicate in vertebrate cells. Viruses.

[5] Libikova H., Heinz F., Ujhazyova D., Stünzner D. (1978). Orbiviruses of the Kemerovo complex and neurological diseases. Med. Microbiol. Immunol..

[6] Malkova D., Holubova J., Kolman J., Marhoul Z., Hanzal F., Kulkova H., Markvart K., Simkova L. (1980). Antibodies against some arboviruses in persons with various neuropathies. Acta Virol..

[7] Mohd Jaafar F., Belhouchet M., Belaganahalli M., Tesh R.B., Mertens P.P.C., Attoui H. (2014). Full-Genome Characterisation of Orungo, Lebombo and Changuinola Viruses Provides Evidence for Co-Evolution of Orbiviruses with Their Arthropod Vectors. PLoS ONE.

[8] MacLachlan N.J., Guthrie A.J. (2010). Re-emergence of bluetongue, African horse sickness, and other orbivirus diseases. Vet. Res..

[9] Schnagl R.D., Holmes I.H. (1975). Electron microscopy of Japanaut and Tilligerry viruses: Two proposed members of the orbivirus group. Aust. J. Biol. Sci..

[10] Centers for Disease Control and Prevention Arbovirus Catalog: Japanaut. https://wwwn.cdc.gov/arbocat/VirusDetails.aspx?ID=207.

[11] Miura T., Kitaoka M. (1977). Viruses isolated from bats in Japan. Arch. Virol..

[12] Zhao G., Krishnamurthy S., Cai Z., Popov V.L., Travassos da Rosa A.P., Guzman H., Cao S., Virgin H.W., Tesh R.B., Wang D. (2013). Identification of Novel Viruses Using VirusHunter—An Automated Data Analysis Pipeline. PLoS ONE.

[13] Kemp G., Le G.G., Karabatsos N., Rickenbach A., Cropp C. (1988). IFE: A new African orbivirus isolated from Eidolon helvum bats captured in Nigeria, Cameroon and the Central African Republic. Bull. de la Soc. de Pathol. Exot. et de Ses Fil..

[14] Ezeifeka G., Umoh J., Ezeokoli C., Ezealor A. (1987). Prevalence of Ife virus infection in wild rodents and birds from Zaria, Nigeria. J. Wildl. Dis..

[15] Ezeifeka G., Umoh J., Ezeokoli C., Gomwalk N. (1989). Serological evidence of Ife virus infection in Nigerian indigenous domestic ruminants. Trop. Anim. Health Prod..

[16] Centers for Disease Control and Prevention Arbovirus Catalog: Ife. https://wwwn.cdc.gov/arbocat/VirusDetails.aspx?ID=187&SID=4.

[17] Butenko A. (1996). Arbovirus circulation in the Republic of Guinea. Med. Parazitol. i Parazit. Bolezn..

[18] Konstantinov O., Diallo S., Inapogi A., Ba A., Kamara S. (2006). The mammals of Guinea as reservoirs and carriers of arboviruses. Med. Parazitol. i Parazit. Bolezn..

[19] Boiro I., Fidarov F., Lomonossov N., Linev M., Bachkirsov V., Inapogui A. (1986). Isolation of the Fomédé virus from Chiroptera, Nycteris nana, in the Republic of Guinea. Bull. de la Soc. de Pathol. Exot. et de Ses Fil..

[20] Justines G., Kuns M. (1970). Matucare virus, a new agent recovered from Ornithodoros (Alectorobius) boliviensis. Am. J. Trop. Med. Hyg..

[21] Attoui H., Mendez-Lopez M.R., Rao S., Hurtado-Alendes A., Lizaraso-Caparo F., Jaafar F.M., Samuel A.R., Belhouchet M., Pritchard L.I., Melville L. (2009). Peruvian horse sickness virus and Yunnan orbivirus, isolated from vertebrates and mosquitoes in Peru and Australia. Virology.

[22] Jordan I., Horn D., Oehmke S., Leendertz F.H., Sandig V. (2009). Cell lines from the Egyptian fruit bat are permissive for modified vaccinia Ankara. Virus Res..

[23] Ivanova N.V., Zemlak T.S., Hanner R.H., Hebert P.D. (2007). Universal primer cocktails for fish DNA barcoding. Mol. Ecol. Notes.

[24] Messing J. (1983). New M13 vectors for cloning. Methods in Enzymology.

[25] Towner J.S., Amman B.R., Sealy T.K., Carroll S.A.R., Comer J.A., Kemp A., Swanepoel R., Paddock C.D., Balinandi S., Khristova M.L. (2009). Isolation of genetically diverse Marburg viruses from Egyptian fruit bats. PLoS Pathog..

[26] Miller B.R., Mitchell C.J., Ballinger M.E. (1989). Replication, tissue tropisms and transmission of yellow fever virus in Aedes albopictus. Trans. R. Soc. Trop. Med. Hyg..

[27] Kading R.C., Gilbert A.T., Mossel E.C., Crabtree M.B., Kuzmin I.V., Niezgoda M., Agwanda B., Markotter W., Weil M.R., Montgomery J.M. (2013). Isolation and molecular characterization of Fikirini rhabdovirus, a novel virus from a Kenyan bat. J. Gen. Virol..

[28] Langmead B., Salzberg S.L. (2012). Fast gapped-read alignment with Bowtie 2. Nat. Methods.

[29] Martin M. (2011). Cutadapt removes adapter sequences from high-throughput sequencing reads. EMBnet J..

[30] Fu L., Niu B., Zhu Z., Wu S., Li W. (2012). CD-HIT: Accelerated for clustering the next-generation sequencing data. Bioinformatics.

[31] Bankevich A., Nurk S., Antipov D., Gurevich A.A., Dvorkin M., Kulikov A.S., Lesin V.M., Nikolenko S.I., Pham S., Prjibelski A.D. (2012). SPAdes: A new genome assembly algorithm and its applications to single-cell sequencing. J. Comput. Biol..

[32] Belhouchet M., Jaafar F.M., Tesh R., Grimes J., Maan S., Mertens P.P., Attoui H. (2010). Complete sequence of Great Island virus and comparison with the T2 and outer-capsid proteins of Kemerovo, Lipovnik and Tribec viruses (genus Orbivirus, family Reoviridae). J. Gen. Virol..

[33] Belaganahalli M.N., Maan S., Maan N.S., Tesh R., Attoui H., Mertens P.P. (2011). Umatilla virus genome sequencing and phylogenetic analysis: Identification of stretch lagoon orbivirus as a new member of the Umatilla virus species. PLoS ONE.

[34] Gouy M., Guindon S., Gascuel O. (2010). SeaView version 4: A multiplatform graphical user interface for sequence alignment and phylogenetic tree building. Mol. Biol. Evol..

[35] Sievers F., Wilm A., Dineen D., Gibson T.J., Karplus K., Li W., Lopez R., McWilliam H., Remmert M., Söding J. (2011). Fast, scalable generation of high-quality protein multiple sequence alignments using Clustal Omega. Mol. Syst. Biol..

[36] Capella-Gutiérrez S., Silla-Martínez J.M., Gabaldón T. (2009). trimAl: A tool for automated alignment trimming in large-scale phylogenetic analyses. Bioinformatics.

[37] Darriba D., Taboada G.L., Doallo R., Posada D. (2012). jModelTest 2: More models, new heuristics and parallel computing. Nat. Methods.

[38] Guindon S., Gascuel O. (2003). A simple, fast, and accurate algorithm to estimate large phylogenies by maximum likelihood. Syst. Biol..

[39] Darriba D., Taboada G.L., Doallo R., Posada D. (2011). ProtTest 3: Fast selection of best-fit models of protein evolution. Bioinformatics.

[40] Ronquist F., Huelsenbeck J.P. (2003). MrBayes 3: Bayesian phylogenetic inference under mixed models. Bioinformatics.

[41] Kumar S., Stecher G., Tamura K. (2016). MEGA7: Molecular evolutionary genetics analysis version 7.0 for bigger datasets. Mol. Biol. Evol..

[42] Le S.Q., Gascuel O. (2008). An improved general amino acid replacement matrix. Mol. Biol. Evol..

[43] Beaty B., Calisher C., Shope R., Schmidt N., Emmons R. (1989). Arboviruses. Diagnostic Procedures for Viral, Rickettsial and Chlamydial Infections.

[44] Schuh A.J., Amman B.R., Apanaskevich D.A., Sealy T.K., Nichol S.T., Towner J.S. (2016). No evidence for the involvement of the argasid tick Ornithodoros faini in the enzootic maintenance of marburgvirus within Egyptian rousette bats (*Rousettus aegyptiacus*). Parasites Vectors.

[45] Belhouchet M., Mohd Jaafar F., Firth A.E., Grimes J.M., Mertens P.P.C., Attoui H. (2011). Detection of a fourth orbivirus non-structural protein. PLoS ONE.

[46] Van Dijk A.A., Huismans H. (1988). In vitro transcription and translation of bluetongue virus mRNA. J. Gen. Virol..

[47] Zeller H., Karabatsos N., Calisher C., Digoutte J.-P., Cropp C., Murphy F., Shope R. (1989). Electron microscopic and antigenic studies of uncharacterized viruses. III. Evidence suggesting the placement of viruses in the familyReoviridae. Arch. Virol..

[48] Karabatsos N. (1985). International Catalogue of Arthropod-Borne Viruses.

[49] Centers for Disease Control and Prevention Arbovirus Catalog: Chobar Gorge. https://wwwn.cdc.gov/arbocat/VirusDetails.aspx?ID=111&SID=5.

[50] Attoui H., Stirling J.M., Munderloh U.G., Billoir F., Brookes S.M., Burroughs J.N., de Micco P., Mertens P.P., de Lamballerie X. (2001). Complete sequence characterization of the genome of the St Croix River virus, a new orbivirus isolated from cells of Ixodes scapularis. J. Gen. Virol..

[51] Shaw A.E., Ratinier M., Nunes S.F., Nomikou K., Caporale M., Golder M., Allan K., Hamers C., Hudelet P., Zientara S. (2013). Reassortment between two serologically unrelated bluetongue virus strains is flexible and can involve any genome segment. J. Virol..

[52] Nuttall P.A., Moss S.R. (1989). Genetic reassortment indicates a new grouping for tick-borne orbiviruses. Virology.

[53] Hölzer M., Krähling V., Amman F., Barth E., Bernhart S.H., Carmelo V.A., Collatz M., Doose G., Eggenhofer F., Ewald J. (2016). Differential transcriptional responses to Ebola and Marburg virus infection in bat and human cells. Sci. Rep..

[54] Perry A.K., Gang C., Zheng D., Hong T., Cheng G. (2005). The host type I interferon response to viral and bacterial infections. Cell Res..

[55] Zhou P., Tachedjian M., Wynne J.W., Boyd V., Cui J., Smith I., Cowled C., Ng J.H., Mok L., Michalski W.P. (2016). Contraction of the type I IFN locus and unusual constitutive expression of IFN-α in bats. Proc. Natl. Acad. Sci. USA.

[56] Pavlovich S.S., Lovett S.P., Koroleva G., Guito J.C., Arnold C.E., Nagle E.R., Kulcsar K., Lee A., Thibaud-Nissen F., Hume A.J. (2018). The Egyptian rousette genome reveals unexpected features of bat antiviral immunity. Cell.

