# Supplementary material for: Discovery and Characterization of Bukakata orbivirus (Reoviridae:Orbivirus), a Novel Virus from a Ugandan Bat
Source: Viruses. 2019 Mar 2;11(3):209. doi: 10.3390/v11030209 (PMC6466370; doi:10.3390/v11030209)
Supplement: Supplementary file 1 [file viruses-11-00209-s001.zip › Fagre_Supplemental/Supplemental Tables and Figure Captions.docx]

**Captions for Supplemental Figures and Tables**

**Supplemental Figure 1 (Fig. S1).** Plaque demonstrating cytopathic effect caused by BUKV on Vero cells derived from splenic homogenate from Bat UGA432.

**
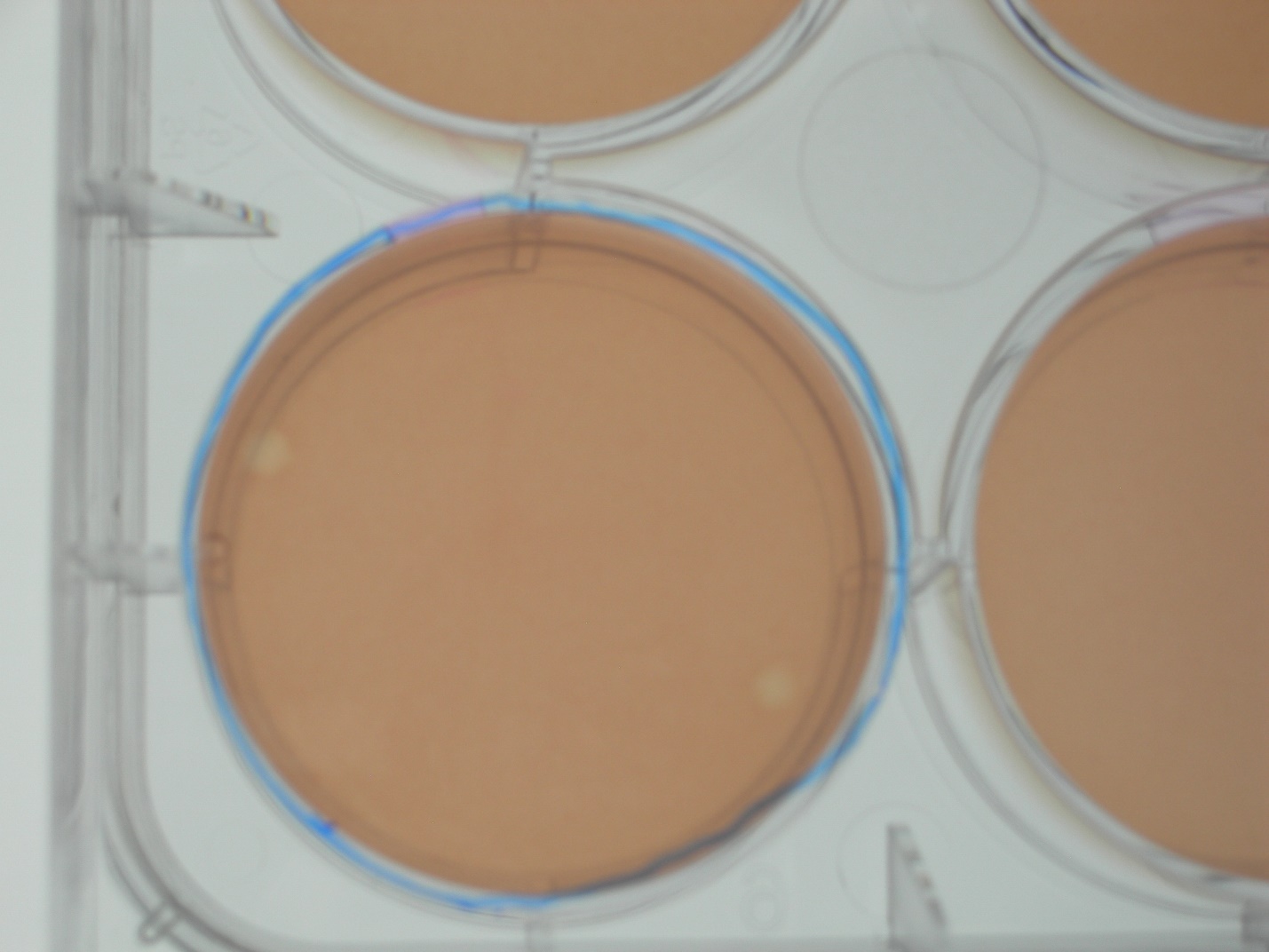
**

**Supplemental Figure 2 (Fig. S2).** Bayesian phylogenetic tree of the viral polymerase gene (VP1) of selected orbiviruses (nucleotide). Branch labels show posterior probability and bar shows substitutions per site.


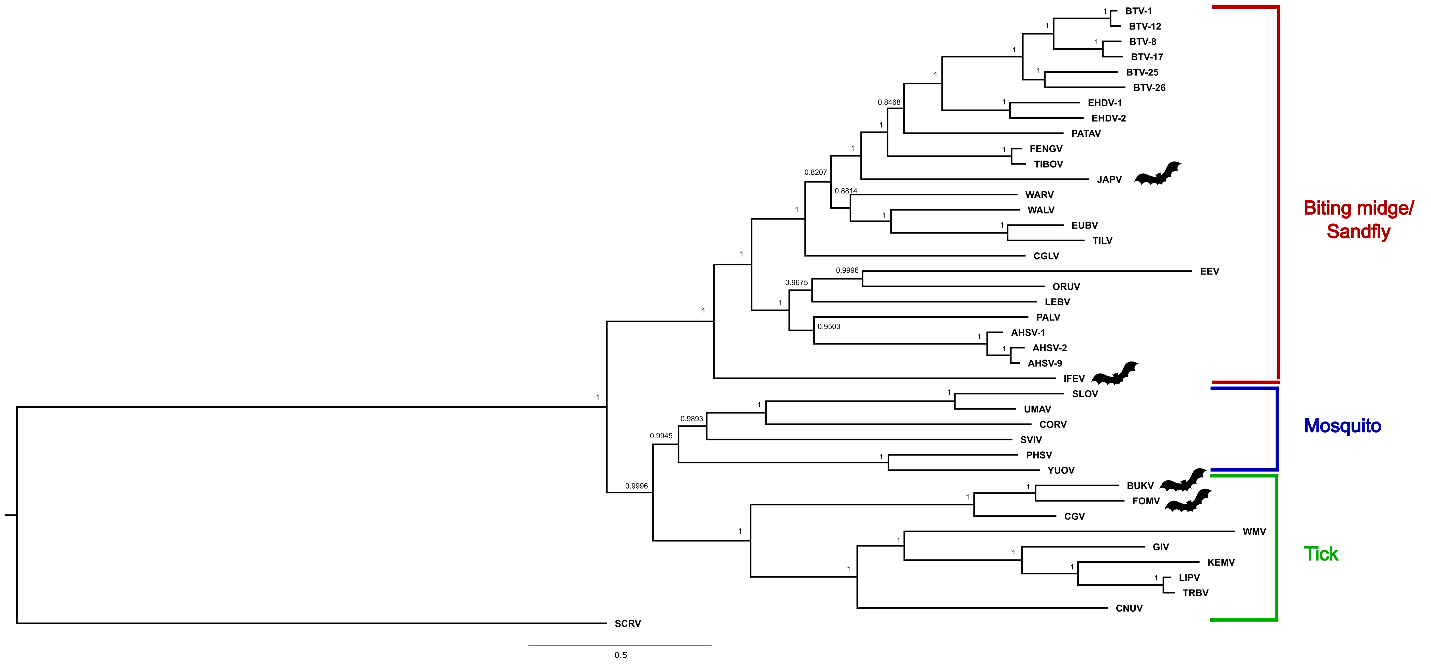


**Supplemental Figure 3 (Fig. S3).** Bayesian phylogenetic tree of the gene encoding sub-core shell (T2) of selected orbiviruses (nucleotide). Branch labels show posterior probability and bar shows substitutions per site.

**
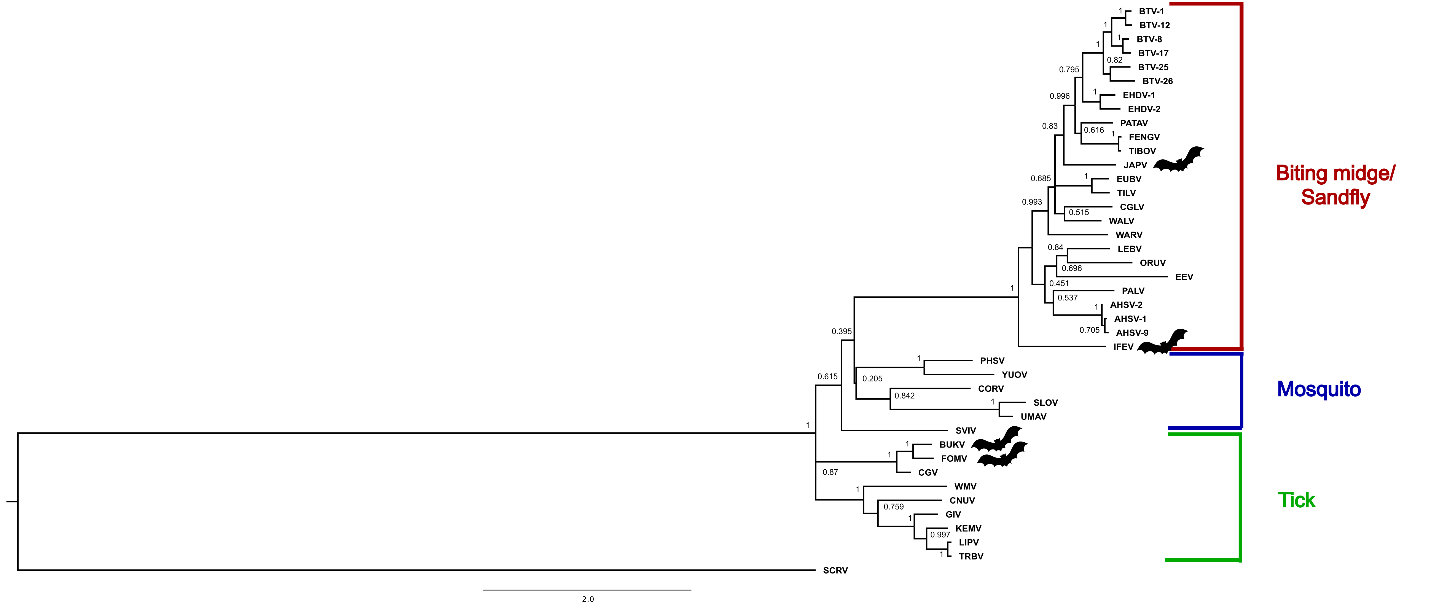
**

**Supplemental Figure 4 (Fig. S4).** Bayesian phylogenetic tree of the gene encoding the outer core protein (T13) of selected orbiviruses (nucleotide). Branch labels show posterior probability and bar shows substitutions per site.

**
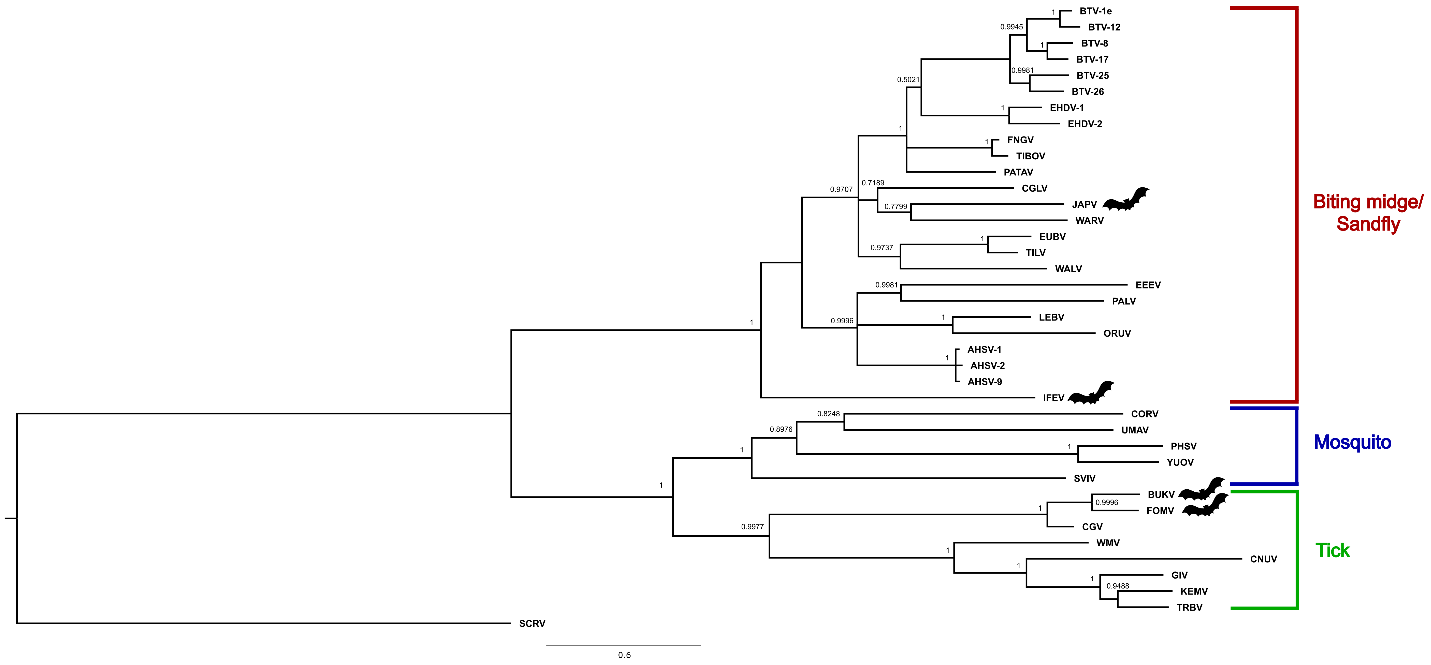
**

**Supplemental Table 1 (Table S1).** Sequences used in phylogenetic analyses of bat-associated orbiviruses.

| **Isolate** | **Abbrev.** | **VP1** | **T2** | **T13** |
| --- | --- | --- | --- | --- |
| AHSV-1 (HS29/62) | AHSV-1 | FJ183364 | FJ183366 | FJ183371 |
| AHSV-2 (strain 2/E.caballus-c/ZAF/2012/Mokopane-E120203) | AHSV-2 | KT030360 | KT030362 | KT030366 |
| AHSV-9 | AHSV-9 | NC_006021 | GQ506549* | U90337* |
| BTV-12 | BTV-12 | GU390658.1 | GU390660 | GU390663 |
| BTV-17 | BTV-17 | L20447 | JX272451* | JX272455 |
| BTV-1e (IND1992/01) | BTV-1 | JQ282770 | JQ282771 | JQ282774 |
| BTV-25 | BTV-25 | GQ982522 | GQ982523 | EU839843* |
| BTV-26 (KUW2010/02) | BTV-26 | JN25516 | HM590643 | HM590644 |
| BTV-8w (NET2006/04) | BTV-8 | AM498051 | AM498053 | AM498057 |
| Changuinola (BE AR 49042) | CGLV | JQ610655 | JQ610657 | JQ610661 |
| Chenuda virus | CHUV | NC_027534 | NC_027535 | NC_027550 |
| Chobar Gorge virus | CGV | NC_027553 | NC_027554 | NC_027559 |
| Corriparta virus | CORV | KC853042 | KC853043 | KC853049 |
| EEV (Cascara) | EEV | HQ630912 | HQ630914 | HQ630918 |
| EHDV-1w (USA1955/01) | EHDV-1 | AM744977 | AM744979 | AM744983 |
| EHDV-2 | EHDV-2 | AM744987.1 | AM744989 | AM744993 |
| Eubenangee virus (AUS1963/01) | EUBV | JQ070376 | JQ070378 | JQ070382 |
| Fengkai (Tibet) | FENGV | NC_027803.1 | NC_027812 | NC_027805 |
| Great Island Virus (CanAr 42) | GIV | HM543465 | HM543466 | HM543471 |
| Kemerovo virus | KEMV | HQ266591 | HQ266592 | HQ266597 |
| Lebombo virus | LEBV | JQ610665 | JQ610666 | JQ610671 |
| Lipovnik (CzArLip 91) | LIPVh | HM543475 | HM543476 | X |
| Orungo virus (UGMP 359) | ORUV | JQ610675 | JQ610677 | JQ610681 |
| Palyam virus | PALY | KT002588 | KT002590 | KT002594 |
| Pata (CAF1968/01) | PATAV | JQ070386 | JQ070388 | JQ070393 |
| Peruvian horse sickness virus | PHSV | DQ248057 | DQ248058 | DQ248063 |
| Sathuvachari (IAn66411) | SVIV | KC432629 | KC432631 | KC432635 |
| St Croix River virus | SCRV | NC_005997 | NC_005998 | NC_006004 |
| Stretch lagoon orbivirus_K49460 | SLOV | NC_012754 | NC_012755 | X |
| Tibet (XZ0906) | TIBV | KF746187 | KF746189 | KF746193 |
| Tilligerry virus (AUS1978/03) | TILV | JQ070366 | JQ070368 | JQ070372 |
| Tribec virus | TRBV | HM543478 | HM543479 | HQ266588 |
| Umatilla virus (USA1969/01) | UMAV | HQ842619 | HQ842620 | HQ842626 |
| Wad Medani virus | WMV | KP268804 | KP268805 | KP268811 |
| Wallal (Ch 12048) - AUS1978/09 | WALV | KJ495745 | KJ495747 | KJ495751 |
| Warrego (Ch 9935) - AUS1969/01 | WARV | KJ495755 | KJ495757 | KJ495761 |
| Yunnan virus | YUOV | NC_007656.1 | NC_007657 | NC_007663 |

**Supplemental Table 2 (Table S2).** Sequence of BUKV VP1 synthetic positive amplification control with altered bases highlighted as compared to the homologous region of the unmodified BUKV VP1.

| **BUKV synthetic positive amplification control** | CGCTCCGTCATTGGTTTGCACTCCTTGTCTCAATCGCGCGTGCGGAGGGCCAACGACCGTTGGGTCTATCCGCAACCTCTGGTCAACTCGTCAATTCCAAGATCGATATATCGCGCGAATCTGCTAAGCTCGAGATCTCGATATCGCTCTATATTAGGATCTCTCTCTAGATCTAGCTATAGCTATCTTTCGATCGATCGAGTTATGCGCGATATCGATCGATACGTTCACGAGAGGAGGGCACAACTGTTTCCCTCATCGTCGACGGTGTCTCTGTGGAGGCTTTGTCGCTCGTGCGTGAGTTCTTCATCGCTTGTCTACCGCATCCAAAGAAAGTATGCAATATGTTGCGTGCAGCGTACACCTGGTTTGTAAAGAACTGGGGGACCGGCGTAAGCGAAGCTAT |
| --- | --- |
| **Unmodified BUKV (bp 380-786)** | CGCTCCGTCATTGGTTTGCACTCCTTGTCTCAATCGCGCGTGCGGAGGGCCAACGACCGTTGGGTCTATCCGCACTTATCGCGTGCATTGACGATCTAGGCGAGCCCTTTCATCAGAATACTCGTGACCTTTCGAAGTATGAAGCCGAGAAGCTATCAAGCTCGATCGTCCTATATGCGGAAATGTGCCTCGCTGAAGCGATACAAGAAATCAATGTGTACTATCGTTCACGAGAGGAGGGCACAACTGTTTCCCTCATCGTCGACGGTGTCTCTGTGGAGGCTTTGTCGCTCGTGCGTGAGTTCTTCATCGCTTGTCTACCGCATCCAAAGAAAGTATGCAATATGTTGCGTGCAGCGTACACCTGGTTTGTAAAGAACTGGGGGACCGGCGTAAGCGAAGCTAT |

**Supplemental Figure 5 (Fig. S5).** Matrix of VP1 pairwise identity percentages of nucleotide (lower left) and amino acid (upper right) between members of the genus *Orbivirus.*


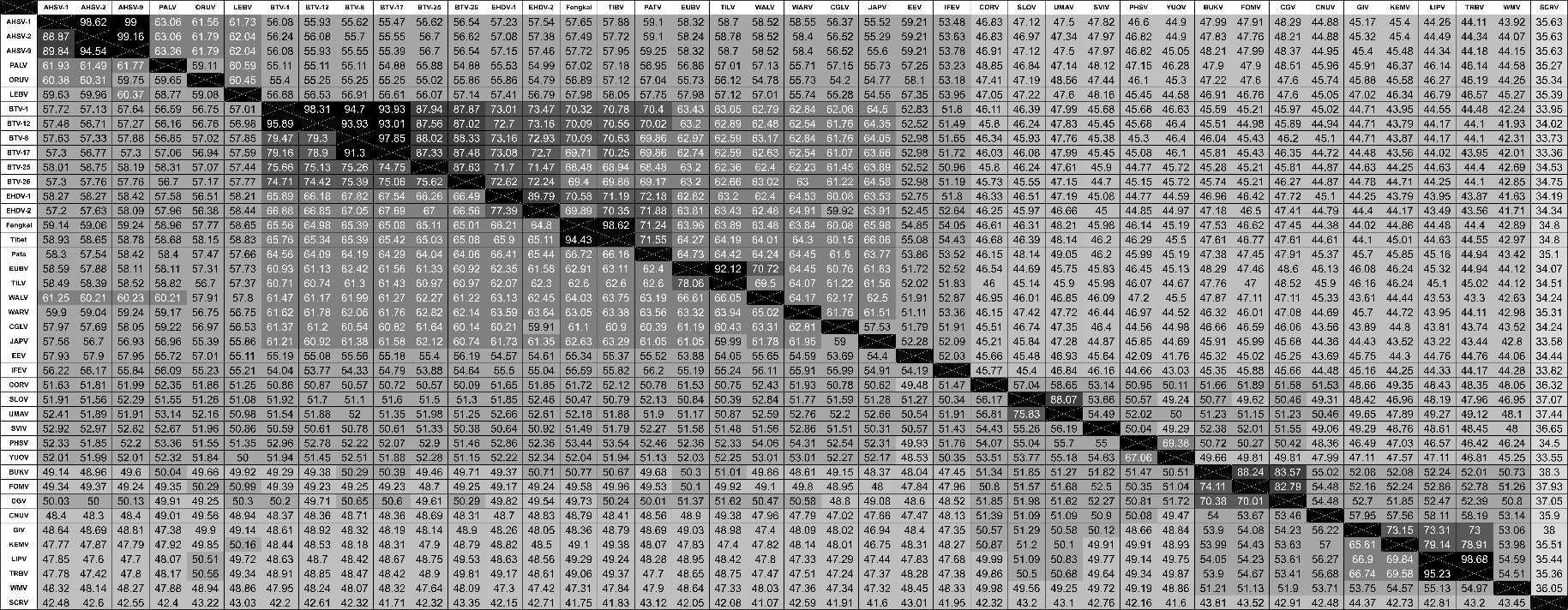


**Supplemental Figure 6 (Fig. S6).** Matrix of T13 pairwise identity percentages of nucleotide (lower left) and amino acid (upper right) between members of the genus *Orbivirus.*

**
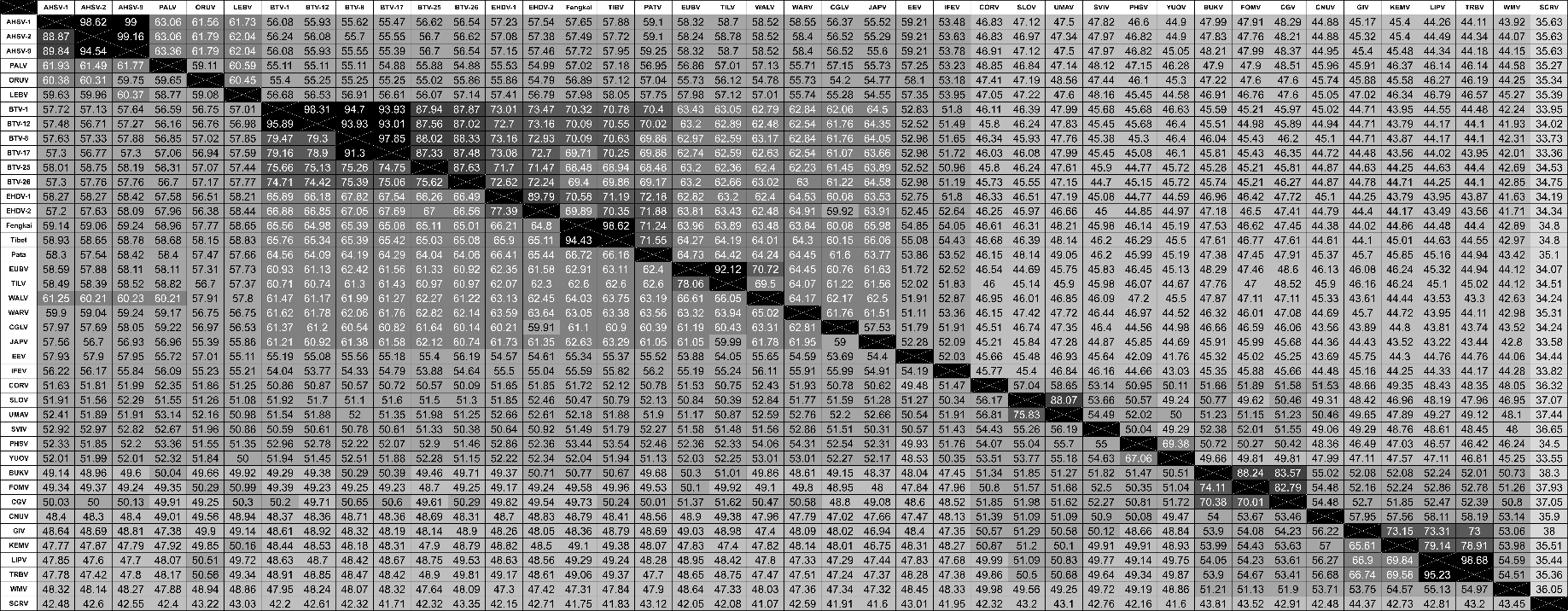
**
