# Supplementary figures and images for: Discovery and Characterization of Bukakata orbivirus (Reoviridae:Orbivirus), a Novel Virus from a Ugandan Bat
Source: Viruses. 2019 Mar 2;11(3):209. doi: 10.3390/v11030209 (PMC6466370; doi:10.3390/v11030209)

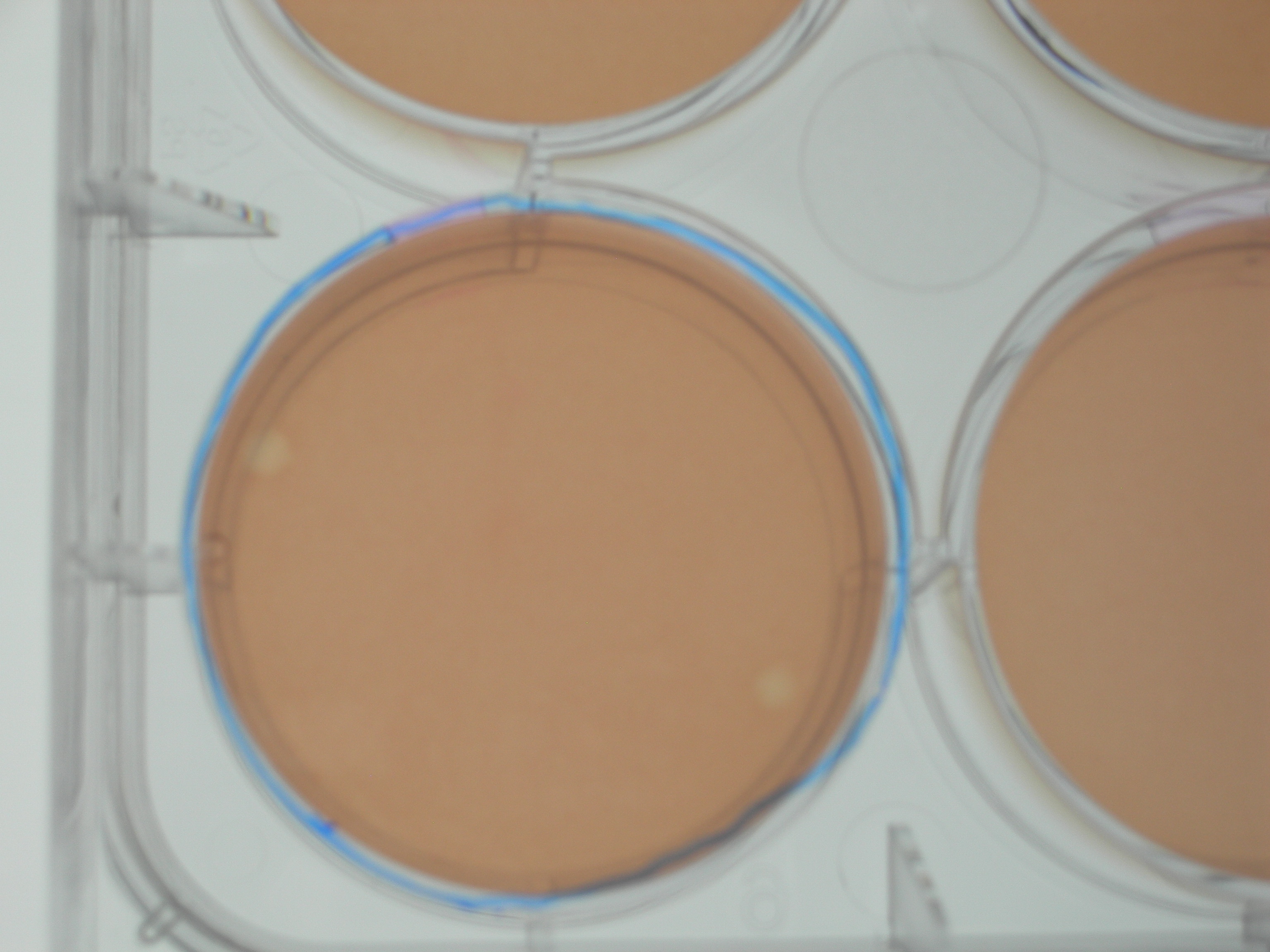

Supplement: Supplementary file 1 [file viruses-11-00209-s001.zip › Fagre_Supplemental/Fig S1. Plaque assay showing BUKV CPE on Vero cells.JPG]

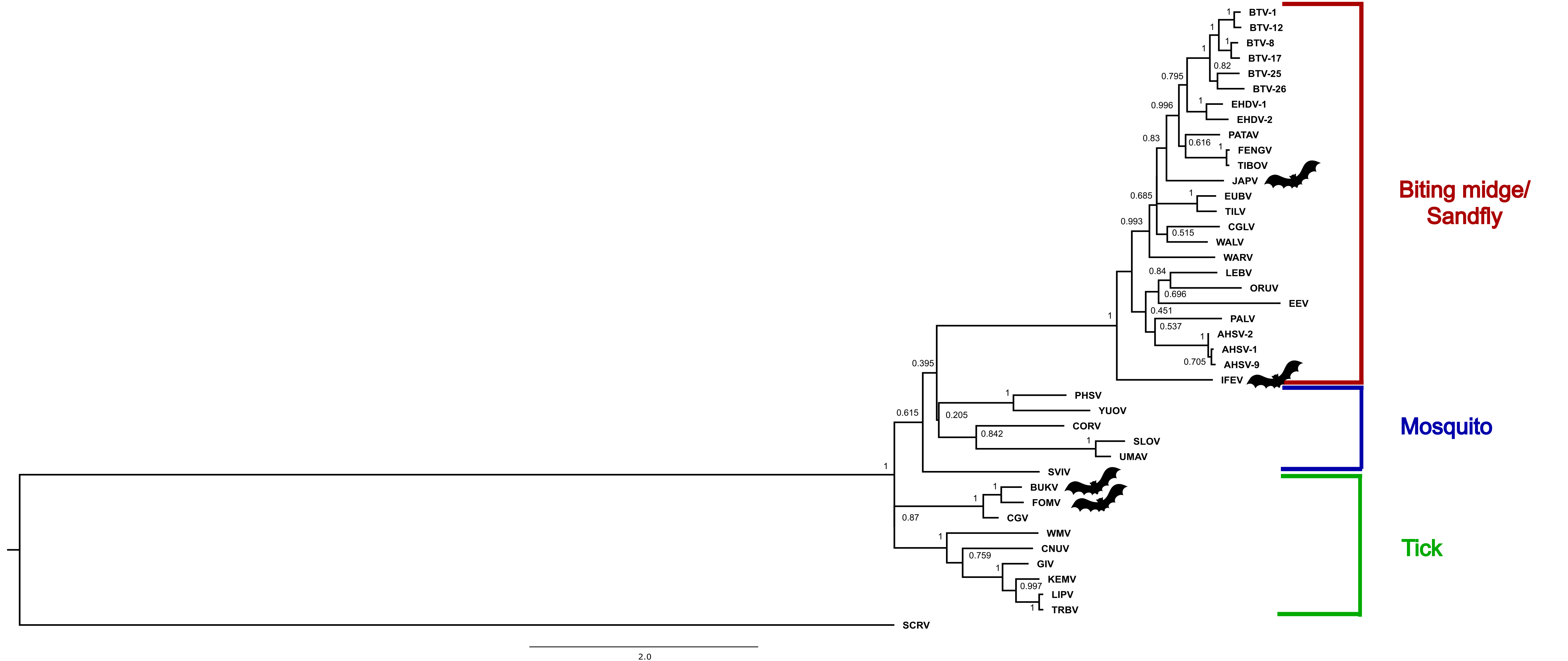

Supplement: Supplementary file 1 [file viruses-11-00209-s001.zip › Fagre_Supplemental/Fig S2. T2 Bayesian NT tree.png]

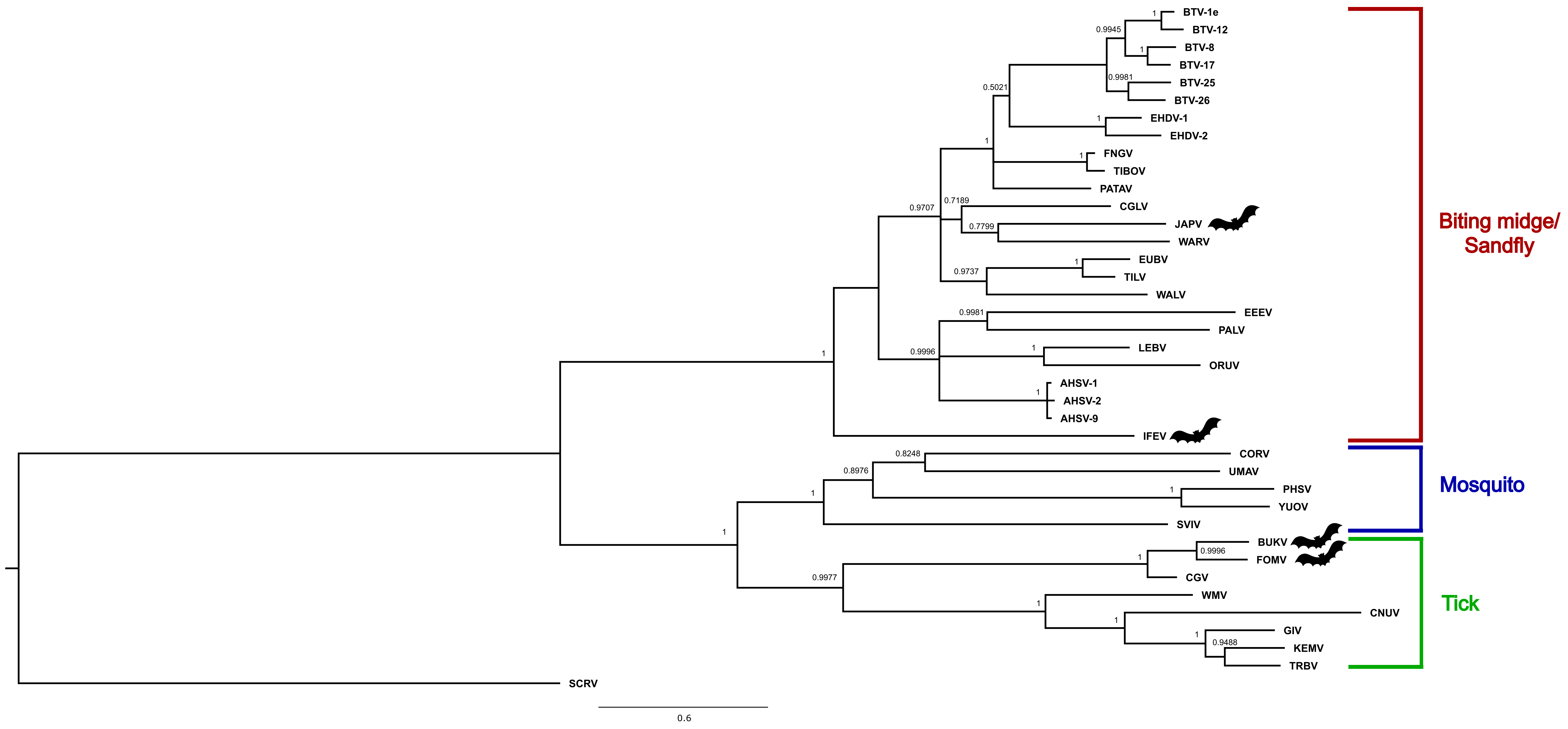

Supplement: Supplementary file 1 [file viruses-11-00209-s001.zip › Fagre_Supplemental/Fig S3. T13 Bayesian NT tree.png]

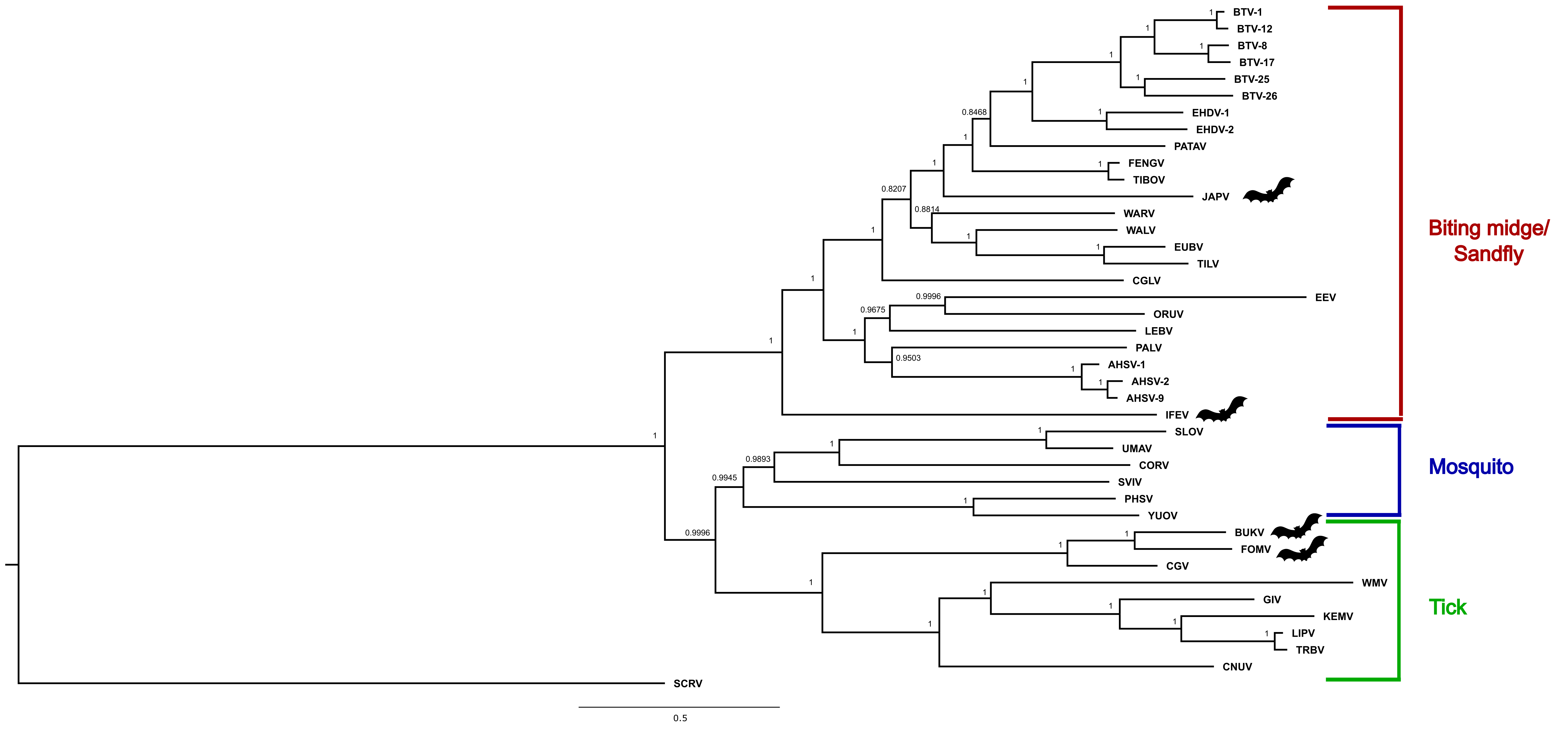

Supplement: Supplementary file 1 [file viruses-11-00209-s001.zip › Fagre_Supplemental/Fig S4. VP1 Bayesian NT tree.png]
